# P-2242. Tick-borne Illness Seroprevalence and Meat Allergy (Alpha-Gal Syndrome): A Series of 15 Cases in Long Island, NY

**DOI:** 10.1093/ofid/ofae631.2395

**Published:** 2025-01-29

**Authors:** Khushbu Shah, Reshma George, Pronoma Srivastava, Michael D Lum, Charles K Vorkas, Brianne Navetta-Modrov, Luis A Marcos

**Affiliations:** Stony Brook University Hospital, Stony Brook, New York; Stony Brook University Hospital, Stony Brook, New York; Stony Brook University Hospital, Stony Brook, New York; Stony Brook University Hospital, Stony Brook, New York; Stony Brook University, Stony Brook, New York; Stony Brook University Hospital, Stony Brook, New York; Renaissance School of Medicine at Stony Brook University, Stony Brook, New York

## Abstract

**Background:**

Alpha-gal syndrome (AGS) is a described meat allergy to an oligosaccharide, galactose-alpha-1,3-galactose (alpha-gal), present on mammalian cells and appears to be associated with the *Amblyomma Americanum* or lone star tick (LST) bite. Suffolk county, NY has an alarmingly high number of cases of AGS. Immunoglobulin E (IgE)-mediated allergic symptomatology has been reported following ingestion of mammalian meat and its by-products though with variable allergy manifestation. The goal of this pilot study was to identify characteristics and clinical manifestations in a cohort of patients with AGS and try to identify possible risk factors for severe manifestations.

**Table 1:**
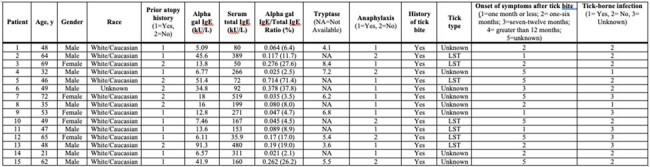
Demographics, atopy history, alpha-gal IgE, serum total IgE and ratios, tick bite history and tick type, onset of symptoms after tick bite, tick-borne infection in patients identified with AGS, N=15. AGS, Alpha-gal syndrome; LST, Lone star tick; IgE, Immunoglobulin E

**Methods:**

A retrospective study was performed to identify adult patients referred to Allergy Clinic at the Stony Brook Hospital between 2018 and 2023 with symptoms and laboratory testing consistent with AGS. Demographics, atopy history, alpha-gal IgE and serum total IgE ratios, allergy manifestation, tick type and tick-borne infections were obtained from chart review.

**Figure 1:**
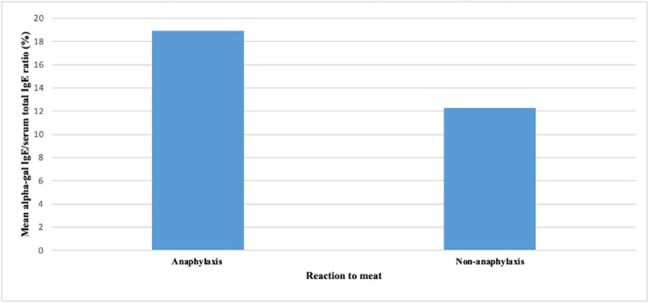
Comparison of mean alpha-gal IgE/serum total IgE ratios in patients with anaphylaxis (N=10) and non-anaphylactic (N=5) manifestations to meat consumption. The mean alpha-gal IgE/serum total IgE ratio in the anaphylaxis group was 18.9% and the mean alpha-gal IgE/serum total IgE ratio in the non-anaphylaxis group was 12.3%. There was no statistically significant difference in mean alpha-gal IgE/serum total IgE ratio between the anaphylaxis and non-anaphylaxis groups (p = 0.5).

**Results:**

We identified 15 adults (mean age: 51 years; male 66.7%; 93% White), with clinical presentation consistent with AGS. Out of the 15 subjects, 9 had history of atopy (60%). All patients reported a history of tick bite, of whom 7 recognized as LST (46.7%). One third of the cases were tested for tick-borne co-infections (n= 10). One met CDC criteria for Lyme disease and 1 had Babesiosis. All patients had elevated alpha-gal IgE/serum total IgE ratio (mean 16.8%, range 2.1-71.4); anaphylaxis group mean ratio was 18.9% versus 12.3% in non-anaphylaxis group (p=0.5). Patients had variability in severity of allergy manifestation: 10 (66.7%) with anaphylaxis, 6 (40%) with skin manifestation, 1 (6.7%) with gastrointestinal symptoms.**Figure 2.** Overview of allergy manifestation reported by patients; anaphylaxis as per AAAAI criteria, N=15.

**Figure:**
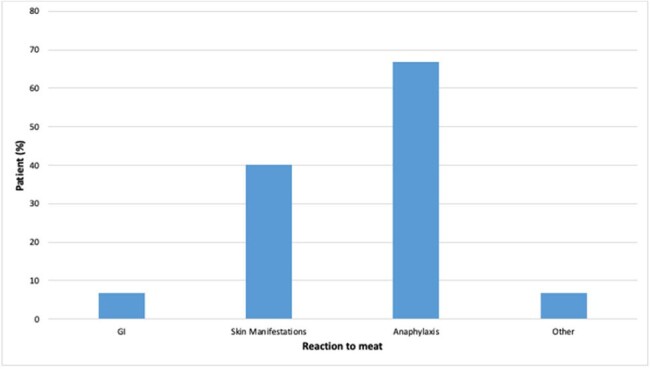
Gastrointestinal; Skin manifestations i.e. urticaria, angioedema, hives; Other i.e itchy throat, eye swelling; AAAA,: American Academy of Allergy, Asthma & Immunology.

**Conclusion:**

Observational data suggests that alpha-gal IgE/total IgE ratio may not be related to disease severity, but the sample size was small. Although, most patients in this cohort did not have a documented history of a previous tick-borne illness despite tick exposure, it is important for Infectious Diseases physicians to be aware of AGS, as many patients are referred after tick bites. Further research regarding risk factors for severe disease is warranted to recognize those at greatest risk to prompt more rapid allergy evaluation.

**Disclosures:**

All Authors: No reported disclosures

